# Georgia State Board of Health

**Published:** 1876-02

**Authors:** 


Georgia State Board of Health.—We have before us the First Annual Report of the Board of Health of the State of Georgia for the year ending October 12, 1875.

While we expected a commencement of the important labors confided to this Board, we must confess to surprise at the amount and value of the material embodied in this publication of 215 pages. In it we find a most concise and able exposition of the objects and labors of the Board in the report of Dr. J. G. Thomas, the President, to the Governor. Next, a highly interesting and practical paper in the report of the Secretary to the Board, in which, among other valuable suggestions for the more efficient working of the Board, we find local or county boards of health recommended as a means of completing the sanitary organization of the State. We also find the most appropriate and forcible address to the medical profession, from the special committee of which Dr. C. B. Nottingham was chairman; most admirably calculated, as was designed, to secure the indispensable co-operation of the physicians of the State. We also have embodied the reports of the two meetings of the Board, in June and October, with copies of the various forms which have been adopted for the returns of registration of births, deaths, and marriages, with a circular of instructions to Ordinaries, and a partial report of vital statistics for the month of August. But among the regular reports of standing and special committees, we find the most imposing array of ability and labor, demonstrating very clearly the wisdom of the Governor in the selection of the members of the board. Here are the reports of the Committees on Endemic, Epidemic and contagious diseases, on the Hygiene of Schools, on the Influence of Trees on Health, on the Sale of Poisons and other Articles Detrimental to Health, on Prisons, and on the most Effectual Means of Preventing Small-Pox in Georgia. We learn from the secretary of the board that the interest in the health law is gradually and decidedly increasing, and that he is sanguine of receiving reports from the entire State in a short period, except, perhaps, the county of Irwin, from which he received the following communication:

" Mr. Taliaferro—*Dear Sir:* Your very kind letter and pamphlet have been received, and I reply for your information, and to let you know my inablity to carry out said law. There is not a physician in the county, nor neither is there any coroner in the county, and the citizens of the county will, to my knowledge, take no interest whatever in any such laws.

"Now, if I am forced to carry out the letter of the law, my first step will be to get rid of my office the best and quickest way possible. The reason we have not got the doctors and officers is, because we have no use for them, and of course such laws will be an injury instead of a benefit to this county.

Your obedient servant,

WILEY WHITLEY,
*Ordinary of Irwin County.*

We regret that our space will not permit us to do more than mention the able report to the Governor, by the president of the board, and the elaborate and excellent reports of the standing committees, but promise in our next number a brief review of these valuable papers.

If the board can be properly sustained by the medical men of the State, the ordinaries, upon whom absolutely depend the success of the measure, will gradually fall into line, and discharge the duties imposed upon them. They should, doubtless, as suggested in this report, be paid some adequate compensation for their services, and yet, in the absence of any provisions of this kind, they should not complain, as it is one of the best paid offices in the counties, and if the returns are promptly recorded, and the reports made monthly to the secretary of the board, the burden will not prove very serious. In conclusion, we know of no feature of our State Government which is fraught with more real and substantial value to the people of the State than the Health Department, if liberally fostered by the Legislature, and properly sustained by the medical profession and those to whom have been entrusted the discharge of duties connected with registration.

That there is opposition to the health law passed at the last session of the Legislature, and especially that portion which provides for registration, cannot be denied, and it is within the limits of possibility that ignorance, prejudice and demagogueism may throw obstacles in the way of its execution as to materially retard, if not altogether destroy, its successful working, but in any event the first volume of Transactions, to which we have already alluded, freighted as it is with the results of labor, talent and benevolence, cannot be blotted out, but will stand as a monument to the intelligence and philanthropy of those who conceived and executed this plan for the advancement of sanitary knowledge in the interest of humanity.

